# Cash stock strategies during regular and COVID-19 periods for bank branches by deep learning

**DOI:** 10.1371/journal.pone.0268753

**Published:** 2022-06-07

**Authors:** Chattriya Jariyavajee, Taninnuch Lamjiak, San Ratanasanya, Suthida Fairee, Kreecha Puphaiboon, Charoenchai Khompatraporn, Jumpol Polvichai, Booncharoen Sirinaovakul

**Affiliations:** 1 Department of Computer Engineering, Faculty of Engineering, King Mongkut’s University of Technology Thonburi, Bangkok, Thailand; 2 Department of Computer and Information Science, King Mongkut’s University of Technology North Bangkok, Bangkok, Thailand; 3 Independent Researcher, Bangkok, Thailand; 4 Data Intelligence Department, Bank of Ayudhya Public Company Limited (Krungsri), Bangkok, Thailand; 5 Department of Production Engineering, Faculty of Engineering, King Mongkut’s University of Technology Thonburi, Bangkok, Thailand; 6 Graduate School of Management and Innovation, King Mongkut’s University of Technology Thonburi, Bangkok, Thailand; Southwest Jiaotong University, CHINA

## Abstract

Determining the optimal amount of cash stock reserved in each bank branch is a strategic decision. A certain level of cash stock must be kept and ready for cash withdrawal needs at a branch. However, holding too much cash not only forfeits opportunities to make profit from the exceeding amount of cash in the stock but also increases insurance cost. This paper presents cash stock strategies for bank branches by using deep learning. Deep learning models were applied to historical data collected by a retail bank to predict the cash withdrawals and deposits. Data preparation and feature selection to identify important attributes from the bank branch data were performed. In the prediction process, two Recurrent Neural Network techniques—Long Short-Term Memory and Gated Recurrent Units methods—were compared. Then prediction errors were measured and statistically tested for their probability distributions. These distributions together with the predicted values were used in determining the lower and upper bounds for holding the cash stock. These bounds were employed to recommend the cash stock level strategies by having two options for different situations. The impacts of COVID-19 were also tested and discussed. According to the bank under this study, the proposed strategies can reduce the amount of cash stock by more than 10% for which was their initial target. Hence, the costs of cash management such as insurance cost and cash transportation cost were reduced. Moreover, the excess cash could be used for other purposes of the bank.

## Introduction

Cash management has always been a challenge for commercial banks. Even with online banking service nowadays, existence of retail branches is still needed in many areas [1]. The amount of cash that should be held at individual branches is not easy to determine. Commercial banks need to keep a certain cash inventory at their branches to serve withdrawal needs. This is because insufficient cash inventory to swiftly serve withdrawal requests may damage the reputation and trust to the banks. On the other hand, holding too much cash incurs opportunity cost in loaning it out to make profit [1–3].

To properly decide the level of cash inventory, the cash withdrawal demands by the customers—even though uncertain—must be predicted. Deep learning (DL) is the newest addition to modern prediction techniques by which historical demand patterns are learned in order to foretell the approaching demands. DL has been applied to many applications such as a chatbot [4], robotics [5], and healthcare [6]. There are also DL technologies integrated in investment [7–9], customer service [10, 11], and marketing in banking and financial services [12, 13]. However, to our knowledge there is no application of DL to manage cash inventory of commercial banks in the literatures. There are only a handful of studies that used DL in predicting cash flow [14] and ATM cash demands to optimize the replenishment transportation schedule [15–17]. A few studies that directly addresses cash stock at the branch level we found are by [1, 3, 18]. However, their approaches differed from ours. Lázaro et al. [18] modelled the cash logistics in bank branches as a robust optimization problem, and devised machine learning to assess cash demand uncertainty that was fed back into the optimization model. Cabello and Lobillo [1] developed the cash demand model as a stochastic process called compound Poisson processes, and then used a mathematical program to minimize associated costs, assuming all those costs could be estimated. Cardona and Morena [3] predicted the cash balances of individual branches using neural network and time-series forecasting techniques. Their predictions were then supplied to a linear program to reduce cash management costs. Other studies were related to banking businesses, but not at the branch level nor on cash inventory.

There are different structures of Deep Neural Network. The most common ones are Convolution Neural Network (CNN), Autoencoder, Restricted Boltzmann Machine (RBM) and Long Short-Term Memory (LSTM) [19]. CNN is a neural network designed for image and video recognition. Autoencoder uses an unsupervised algorithm. It learns the representation in the input data set for dimensionality reduction and recreating the original data set. RBM applies an unsupervised learning algorithm to build non-linear generative models from unlabeled data [20]. Both Gated Recurrent Units (GRU) and LSTM were developed from RNN. These DL techniques utilize an encoder-decoder architecture by which update gates are added to GRU. Likewise, memory and forget gates are included in LSTM to recognize patterns pertaining to the data. LSTM has advantages in managing time-series data by adding memory gates to remember previous input. The memory gates help LSTM to perform more effectively for predicting time series data [21]. Meanwhile, GRU simplifies the memory gates in LSTM [22].

There is another DL technique with a good performance in sequential forecasting called Transformer. The technique also utilizes the encoder-decoder architecture in which the input and its positions are encoded while the output and its positions are decoded before training [23]. Transformer solves problems with a large amount of data. However, Ezen-Chan [24] found that the technique was outperformed by LSTM when it was operated on a small dataset.

In this research, the daily amounts of cash withdrawals and deposits are time-series and the daily dataset started from 2018 until 2020. There are roughly 1,000 records which are considered as a small dataset. Therefore, GRU and LSTM techniques were tested and compared in the experiments.

The organization of this research is as follows. First, DL techniques were applied to the data collected by a commercial bank in Thailand to predict the cash withdrawals and deposits. The number of days per week that the branch was closed was tested to check if it had any effect to customers’ decision to withdraw or deposit cash prior to or right after those days off. Prediction errors were then estimated through statistical distributions. The distributions addressed uncertainty and risk tolerance that the bank was willing to take, and led to establishment of the cash safety stock. Finally, practical considerations were discussed, and they were incorporated to adjust the level of cash stock. This approach was deemed suitable to the bank we worked with because the bank was willing to sacrifice certain prediction accuracy over less intensive data-collection requirement. They were also more comfortable to have flexibility in switching among various cash stock strategies since the behavior of the customers and bank’s policies could change over time. They also needed some time to adjust to this new DL-assisted practice to cash stock management. The impacts of COVID-19 were discussed where it was applicable.

## Methodology

The total daily amounts of cash deposited, withdrawn, and net cash at the end of the day are to be referred as cash in (CI), cash out (CO), and cash stock (CS) attributes. The CI attribute is aggregated from the cash deposit transactions at the end of the day which consists of less than, as well as equal to and greater than one million Baht. Similar calculation is performed to the CO attribute for cash withdrawn. The CS attribute is the total amount of cash remained at the end of the day calculated by adding the cash in different denominations of the banknotes as shown in Table 1.

**Table 1:** Aggregate attributes.

Aggregate attribute	Description
CI	The total amount of cash deposited by customers
	(CASHIN_AMT + CASH_IN_AMT_GT1M)*
CO	The total amount of cash withdrawn by customers
	(CASH_OUT_AMT + CASH_OUT_AMT_GT1M)*
CS	The total amount of cash remained at the end of the day
	(CASHSTOCK1K + CASHSTOCK500 + CASHSTOCK100 + CASHSTOCK_OTHER)*

* The description of each parameter is given in S1 Table.

**Fig 1:**
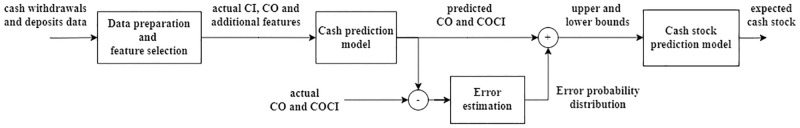
Proposed cash stock prediction methodology.

The methodology proposed in this study is composed of four main processes as shown in Fig 1. First, the data preparation and feature selection extracts important data attributes and data features from the raw data such as cash withdrawals and deposits, the day of the week, the classifications of bank branches, and so on, to be used as inputs by the DL model. Second, the cash prediction model predicts the 14-day CO using CI, CO, and the net cash withdrawals minus deposits (CO–CI, or to be referred as COCI) data from the previous 30 days. The LSTM and GRU are deployed as the prediction method. The design of the neural network architecture and its parameters are also experimented in this process. Third, the error estimation calculates the differences between the predictions and the actual data, and then assesses their probability distributions. The upper and lower bounds of CO and COCI are given after this process. Finally, the cash stock prediction determines the expected cash stock from the lower and upper bounds as the cash stock strategies. Details of each process are provided in subsequent sections.

### Data preparation and feature selection

The dataset in this research was provided by Bank of Ayudhya Public Company Limited in Thailand. The bank collected 680,692 end-of-day records from 628 branches between 2018 and 2020 without showing any detail of individual transactions. Apart from date and branch identifier, each record consists of the total amounts of cash deposited and withdrawn by the customers, the amounts of cash shipped in and out by the cash center of the bank, and the amount of cash remained at the end of the day. All attributes and descriptions of the dataset are shown in S1 Table.

The amount of cash on hand at the end of the day—referred as the cash stock—is the available cash at the beginning of the next day to serve all cash transactions during the day. The total cash deposited and withdrawn by the customers influences the cash stock level of the branch. The amounts of cash shipped in and out are upon the request by the branch manager to replenish and deplete the cash stock.

Based on the working days, the branches of the bank can be categorized into three groups as shown in Table 2.

**Table 2:** The number of branches and records grouped by working days.

Working days	Number of branches	Number of records
Every day	128	191,552
Monday–Saturday	13	14,244
Monday–Friday	435	474,796
Total	628	680,592

There are many *attributes* in the data that need to be identified and selected as the data *features* to be learned by the deep learning model. This selection is to save time and improve the learning process. Six relevant attributes are selected as the data features: days of the week, weekends, holidays, weekends and holidays, the number of consecutive days off, and the amount of CI or CO in the top quartile of each branch.

**Fig 2:**
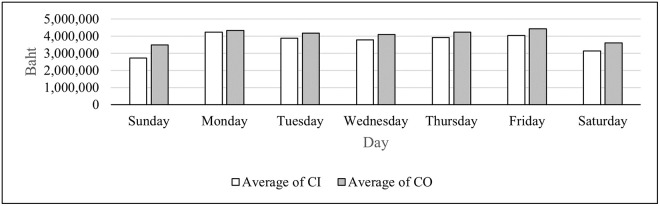
Average CI and CO for branches that open every day.

**Fig 3:**
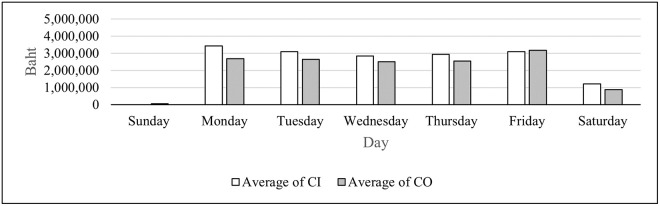
Average CI and CO for branches that open Monday—Saturday.

**Fig 4:**
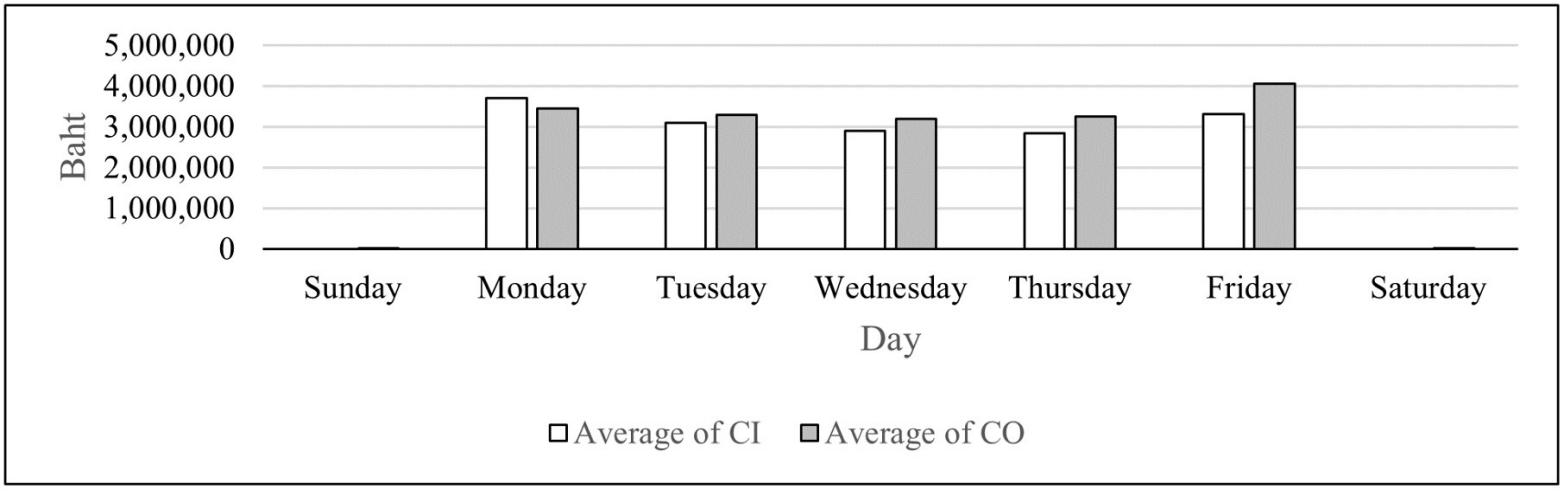
Average CI and CO for branches that open Monday—Friday.

We can categorize the branches based on their operating days in to three groups as shown in Figs 2–4. Of all the branches which open every day in Fig 2, the average CO is higher than that of CI on all seven days. For branches that open from Monday to Saturday in Fig 3, the only day that the average CO is higher than that of CI is Friday. For the branches that open only on weekdays in Fig 4, the average CI is higher than that of CO only on Monday. As seen from these figures, the days of the week impact the average CI and CO, and therefore this attribute is included as a feature for the DL model.

From Figs 2–4, the highest average and the second highest average of CI of all three groups are on Monday and Friday, respectively. The highest average and the second highest average of CO of all three groups are on Friday and Monday, respectively. These results suggest that the amounts of cash deposited and withdrawn right before and right after weekends differ than those of the other days. Therefore, weekends and holidays seem to have an important role in CI and CO; so they become features in the DL model. There are also other annual holidays throughout the year. It was suspected that the number of consecutive days off may alter withdrawal and deposit behavior of the customers. Thus, this attribute is selected as one of the features. Table 3 illustrates how the number of consecutive days off feature is computed.

**Table 3:** Example values of selected features.

#	Feature	April 2018									
		Mon	Tue	Wed	Thu	Fri	Sat	Sun	Mon	Tue	Wed
		9	10	11	12	13	14	15	16	17	18
1	Days of week	2	3	4	5	6	7	1	2	3	4
2	Weekend	1	1	1	1	1	0	0	1	1	1
3	Holiday	1	1	1	1	0	1	1	0	1	1
4	Weekend & Holiday	1	1	1	1	0	0	0	0	1	1
5	Number of consecutive days off										
	• Right before	0	0	0	4	0	0	0	0	0	0
	• Right after	2	0	0	0	0	0	0	0	4	0
	• Right before & right after	2	0	0	4	0	0	0	0	4	0
6	Top 25 percentile										
	• CI	1	0	0	0	0	0	0	0	0	0
	• CO	1	0	0	0	0	0	0	0	0	0

**Fig 5:**
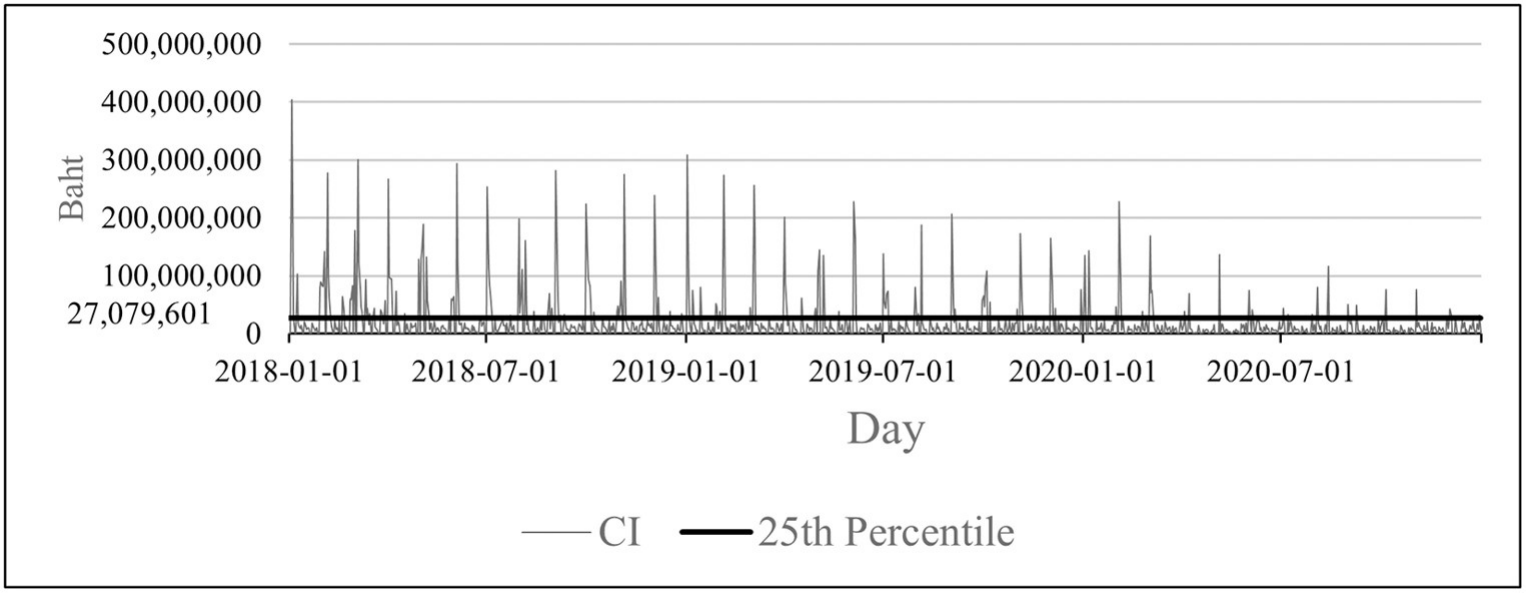
Daily CI in comparison to the 75th percentile of a branch.

Further examination into the data, there are certain days that the amounts of CI or CO are higher than the rest. Fig 5 shows the daily CI of a branch. The days in which the amounts of CI or CO are in the top 25 percentile, to be referred as *top 25 percentile*, are flagged in the DL model to test if this feature affects the predictions. For example, the days that the CI exceeded 27,079,601 Baht which corresponded to the 75th percentile of daily CI was set to 1. Table 3 shows examples of how the top 25 percentile is represented.

Two versions of features were tested. Version 1 consisted of days of week, weekends, holidays, and weekends and holidays. Version 2 included all features of version 1 as well as the number of consecutive days off, and the top 25 percentile. These versions are going to be referred to later on.

#### Cash prediction model

The CO and COCI reflect the amount of cash needed by the bank customers. Since the available data from the bank were daily data, the CO would represent the maximum amount of cash needed by the customers on that day. The COCI, on the other hand, represents the net amount of cash needed on the day.

In this phase, the data of previous 30 consecutive days were used as inputs on a rolling time window to predict CO and COCI of the next 14 days. Each time window consisted of the 30-day data of six features. Then the time window rolled for one day and another set of 30-day data was presented as the inputs. Specifically, the data of days 2 to 31 were in this second time window. The time window kept rolling, one day at a time, until the end of the training data. The data from the bank were simply daily CO and CI of each of the branches. Thus, a pre-processing procedure was required. The outputs or the predictions were rolling in a similar fashion. For example, in the predictions of the first 14-day represented the CO and COCI for days 31 to 44. Then in the next set of outputs the predictions were the CO and COCI for days 32 to 45, and so forth. The time window of 30-day input data was selected to represent monthly cash demands since certain customers behave according to their monthly salary payment and billing cycle. The 14-day time window of the outputs (predictions) was chosen to coincide with the cash delivery cycle for which it was planned two weeks in advance. In other words, the cash delivered in each cycle should cover the cash demands of that branch for two weeks to minimize the delivery cost.

**Fig 6:**
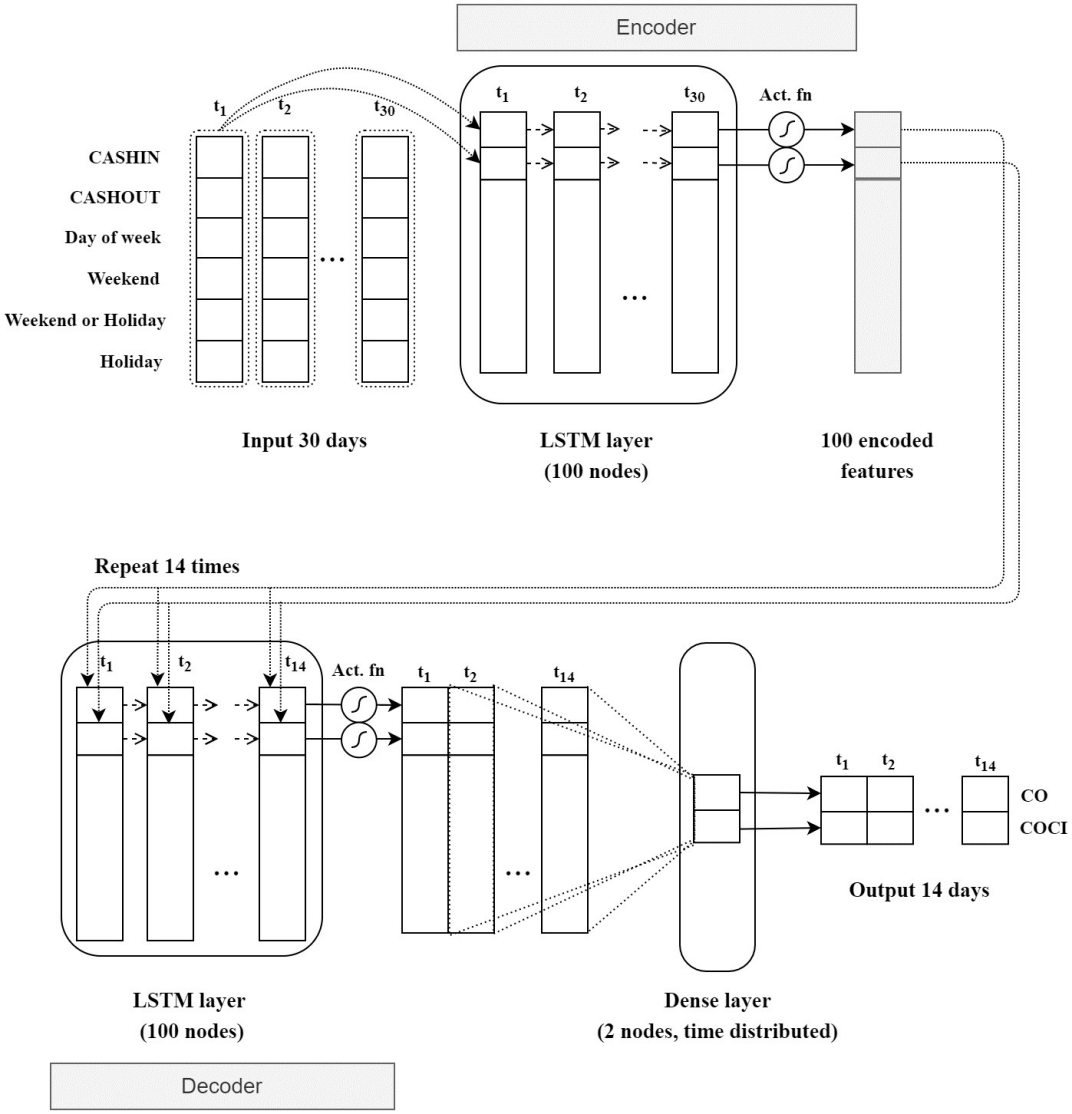
Cash stock prediction model structure based on LSTM algorithm.

The structure of prediction model based on Long Short-Term Memory (LSTM) is shown in Fig 6. It consists of multiple sequentially connected neural network layers. In this research, LSTM [21] and GRU [22] were used interchangeably in the structure. Since the outputs of the models were the prediction values for the next 14 days, encoder and decoder techniques were applied. The encoder technique was executed to reduce the input dimension of 30 days to a one column vector. Then the vector was copied 14 times for the 14-day outputs. LSTM was deployed to predict CO and COCI. There were two layers of LSTM, each with 100 neurons. Each layer determined whether the inputs should be retained in its memory. Hyperbolic Tangent Activation Function (tanh) [25] was exercised as the activation function to cope with output value normalization after each LSTM layer. The outputs from the decoder were connected to a dense layer to create CO and COCI predictions. In model training, Adam [26] was adopted as the optimizer of the models because of its ability to avoid local minima with adaptive estimation of first-order and second-order moments. According to [26], Adam was robust and well suited to a wide range of non-convex optimization problems in the field of machine learning. The structure of GRU based prediction model was similar to that of LSTM, but just replacing of LSTM by GRU. The LSTM and GRU libraries as well as other components from Keras [27] were utilized to operate the model.

### Error estimation

The prediction values from the DL algorithm obtained give us a sense of what the actual values in the near future would be. However, no prediction is perfect, and actual results are likely to differ from the predicted ones. This is due to randomness regardless of the perfect prediction process [28]. It is thus important to take into account the prediction errors.

To deal with errors, they need to be described and estimated. In DL, the errors are often quantified by root mean squared error (RMSE) values. The prediction methods that offer low RMSE are usually the preferable ones. However, to determine the cash stock level at a bank branch, it is important to also know if it is likely that the branch would be understock or overstock. An error measure that offers such indication is a simple error calculation as in the Eq (1).

$$E_{t}=A_{t}-P_{t}$$ (1)

where *t* is the time index, and *E*_*t*_, *A*_*t*_, and *P*_*t*_ denote error value, actual value, and predicted value at time *t*, respectively.

Since there are many data points, so as the error values, it is then more convenient to describe the errors through distributions. Using statistical testing, the distribution that best fits with the error values can be selected as the representative. Errors also imply uncertainty nature of the demands for which the cash stock level must take in to account. While the prediction from DL presents the expected value of the customer demands, there are still chances that certain demands are not served if the cash stock reserved is exactly at the predicted demand level. In practice, it is advisable to carry more inventory (cash in this case) to cope with the risk of upsurge in demands. Even though a higher level of cash stock offers a higher service level to the customers, too much cash stock on hand becomes an opportunity loss to make profit out of the excess cash. It is a managerial decision to determine a suitable cash stock level that represents the willingness to reserve the cash inventory to deal with the demand uncertainty risk. The amount of inventory kept on hand to allow for uncertainty in the demands is called safety stock [29]. Then the total on hand inventory can be calculated from Eq (2) whose safety stock is derived from Eqs (3) and (4).

$$V_{t}=P_{t}+SS$$ (2)

$$Prob\left(E\geq SS\right)=\alpha$$ (3)

$$SS=F_{E}^{-1}(1-\alpha )$$ (4)

where *V*_*t*_ and *P*_*t*_ denote the inventory level and predicted value at time *t*, respectively. Let *SS* be the amount of safety stock which can be calculated from Eq (4), where *α* is an acceptable risk level due to demand uncertainty, *E* is the random variable of the error distribution, and $F_{E}^{-1}$ is the inverse of the cumulative probability function of variable *E*.

### Cash stock prediction model

**Fig 7:**
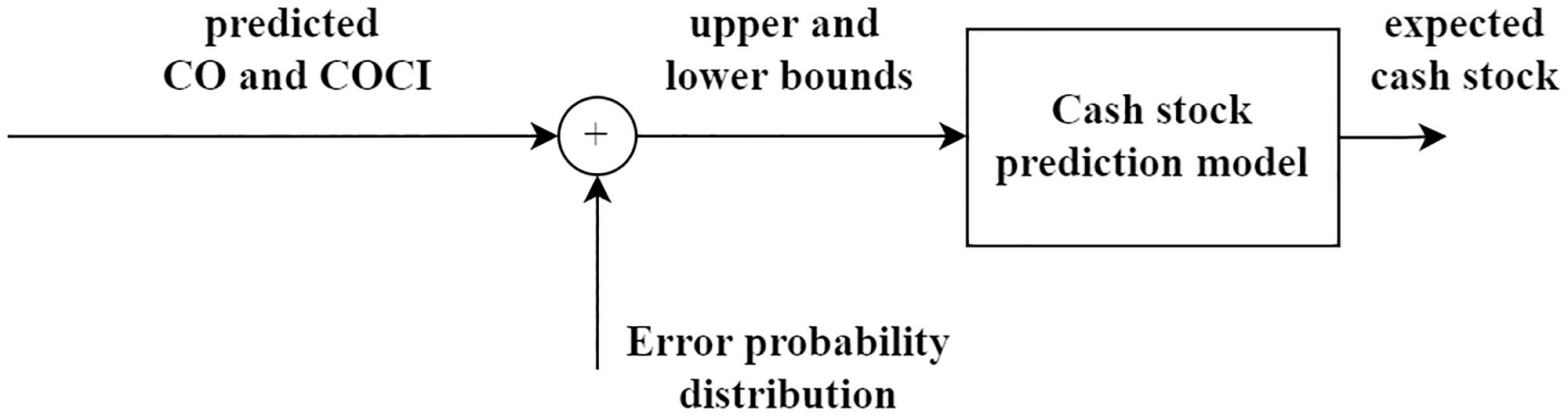
Diagram of cash stock prediction model.

To determine the cash stock level, several considerations are involved. First, the maximum value of cash stock (upper bound) could be estimated from the predicted CO and SS. These values are addressing only the cash demands from the withdrawals. The minimum value of cash stock (lower bound) could be found from the predicted COCI and the associated SS value. Note that this minimum value already accounts for the cash deposits through CI. Fig 7 is the diagram of cash stock prediction model, illustrating how the cash stock prediction is modelled.

To set the upper and lower bounds, the values of CO, COCI, and their prediction errors need to be discussed. The value of the predicted CO must be equal to or greater than zero. The only case that the CO value is zero is when there is no withdrawal at all. The prediction errors on the other hand can be negative, zero, or positive because they are calculated from the predicted values against the actual ones. Hence, the upper bound (UB) should be at least equal to zero; otherwise it should be equal to the sum of the predicted value and the SS as in Eq (5).

The COCI values show the net daily cash demands by which the cash demands are deducted by the cash deposits to calculate COCI. If the cash stock level is established based on these COCI values, the cash needed to be held to serve the customers would be lower than that set by the CO values alone. The bounds obtained by using the CO and COCI values are referred as the upper bound (UB) and the lower bound (LB), respectively. These bounds are calculated by Eqs (5) and (6), orderly.


$$UB=max(0,{Predicted}_{CO}+{SS}_{CO})$$ (5)

$$LB=max(0,{Predicted}_{COCI}+{SS}_{COCI})$$ (6)

A practical cash stock level should be set between these bounds. After consulting with the bank staff, a couple approaches to adjust practical cash stock level are proposed. These approaches are referred as options 1 and 2, and are computed by Eqs (7) and (8), respectively.

The first option is to balance the value of the expected cash stock with the upper and lower bounds by a certain ratio (*r*_1_). By Eq (7), the branch manager is allowed to alter the expected cash stock between the upper and lower bounds by controlling the *r*_1_ ratio. The value of *r*_1_ is between 0 and 1. When the value of *r*_1_ is zero it implies that the expected cash stock is based only on the lower bound. On the contrary, when *r*_1_ = 1 the expected cash stock relies purely on the upper bound. However, the expected cash stock calculated by this option is limited by the minimum threshold (*θ*). This threshold is the lowest amount of cash stock to be kept at the branch. The threshold can be set by the branch manager to complement his/her experience with the DL results.


$$ExpectedCashStockOpt1=max(\theta ,LB+r_{1}\times (UB-LB))$$ (7)

The second option is more conservative as it is computed from the upper bound alone. This option implies that the expected cash stock level is set purely based on the actual withdrawal demand. In this option, the expected cash stock level is a multiple of the upper bound as in Eq (8).

$$ExpectedCashStockOpt2=r_{2}\times UB$$ (8)

where *r*_2_ is the multiplying factor and its value should be at least 1.0.

Eqs (7) and (8) provide the branch manager with flexibility in deciding the cash stock level that agrees with the conditions of the branch. The lower bound in Eq (6) considers both CI and CO, while the upper bound in Eq (5) is calculated from CO only. However, in some branches the CI values are often higher than the CO values. Therefore, if only the CO is considered, we may not be able to effectively reduce the cash stock level of that branch. To resolve this problem, both CI and CO must be considered together, and this becomes Eq (7).

## Experiment

Since April of 2020, Thailand started to observe a substantial rise in the number of COVID-19 cases even though the first COVID-19 patient was found in January of the same year. Several public safety measures against the outbreak had been enforced to prevent further transmission of the disease. Hence, the data in 2020 is impacted by the COVID-19 pandemic. Examples of those measures are closure of public places, schools, and universities [30], a nationwide curfew [31], and a temporary ban of international incoming passenger flights to Thailand [32]. To capture the effect of COVID-19, the model was trained and tested on three time periods as follows.

- *Regular period*: January 2018 –December 2019 (The training and testing data were from January 2018 to June 2019, and July 2019 to December 2019, respectively.)
- *COVID-19 period*: April 2020 –December 2020 (The training and testing data were from April 2020 to October 2020, and November 2020 to December 2020, respectively.)
- *Entire period*: January 2018 –December 2020 (The training and testing data were from January 2018 to March 2020, and April 2020 to December 2020, respectively.)

To test the prediction models, 10 branches were sampled from 628 branches. These sampled branches located in different areas such as downtown, countryside, shopping malls, university campus, and tourist attraction locations. Four of them (Branch A1-A4) are opened every day. Branches B1-B2 operate from Monday to Saturday. The other four branches (Branch C1-C4) work only on weekdays. Every group contains a mix of different areas where the branches locate. The branches in shopping malls or close to a tourist attraction usually open every day. The downtown locations normally close during the weekends. Branches in a university campus and in a downtown commercial area operate every day except Sunday.

### Prediction error measurements

RMSE and mean error (ME) were applied to evaluate the performance of the cash stock prediction model. RMSE can be calculated by Eq (9), and *E*_*t*_ represents the difference between the actual value and the predicted one calculated by Eq (1), where *n* is the number of data points. The model that provides the minimal RMSE value is the best prediction model [33].


$$RMSE=\sqrt{\frac{1}{n}\sum_{t=1}^{n}{\left(E_{t}\right)}^{2}}$$ (9)

To determine whether the model overestimates or underestimates the prediction values, ME is utilized to adjust the model and it can be calculated from Eq (10).


$$ME=\frac{1}{n}\sum_{t=1}^{n}\left(A_{t}-P_{t}\right)$$ (10)

### Experimental results

The RMSE values of the CO and COCI prediction models are shown in Tables 4 and 5. To evaluate the CO prediction models, six variations of the DL model were tested based on the input features (versions 1 and 2 explained earlier) and the period of the training data as listed in Table 4. The activation function used in these variations is tanh. The table shows the RMSE values of each variation. The inputs to this model contained 30 consecutive days of historical data in a one-day rolling time window to predict the CO values for the next 14 days. The results reveal that the combination of version 1 features (v1), tanh activation function, and the training and testing data sets covering the entire period provided the smallest mean and minimum RMSE values among the 10 branches (as highlighted). The same combination of input features also gave the best overall results for the COCI models as shown in Table 5.

**Table 4:** RMSE values of CO results.

Additional feature	RMSE					
	v1	v2	v1	v2	v1	v2
Period	Regular	Regular	Covid-19	Covid-19	Entire	Entire
Branch A1	0.141	0.185	0.158	0.165	0.149	0.154
Branch A2	0.198	0.218	0.171	0.170	0.152	0.158
Branch A3	0.316	0.138	0.254	0.133	0.127	0.129
Branch A4	0.229	0.125	0.189	0.130	0.129	0.133
Branch B1	0.228	0.143	0.219	0.137	0.118	0.129
Branch B2	0.206	0.128	0.142	0.140	0.130	0.127
Branch C1	0.240	0.161	0.253	0.159	0.167	0.176
Branch C2	0.174	0.192	0.174	0.200	0.201	0.212
Branch C3	0.151	0.123	0.136	0.128	0.118	0.120
Branch C4	0.199	0.140	0.199	0.140	0.135	0.143
Mean	0.209	0.157	0.188	0.151	**0.143**	**0.148**
Minimum	0.141	0.123	0.136	0.128	**0.118**	**0.120**

**Table 5:** RMSE values of COCI results.

Additional feature	RMSE					
	v1	v2	v1	v2	v1	v2
Period	Regular	Regular	Covid-19	Covid-19	Entire	Entire
Branch A1	0.079	0.078	0.065	0.054	0.086	0.053
Branch A2	0.111	0.113	0.165	0.159	0.090	0.097
Branch A3	0.141	0.146	0.158	0.138	0.103	0.100
Branch A4	0.108	0.117	0.126	0.138	0.086	0.077
Branch B1	0.062	0.065	0.125	0.134	0.042	0.042
Branch B2	0.100	0.115	0.137	0.126	0.099	0.087
Branch C1	0.141	0.147	0.197	0.201	0.149	0.155
Branch C2	0.106	0.100	0.066	0.068	0.063	0.095
Branch C3	0.073	0.072	0.115	0.119	0.059	0.056
Branch C4	0.508	0.125	0.163	0.176	0.072	0.127
Mean	0.143	0.108	0.132	0.131	**0.085**	**0.089**
Minimum	0.062	0.065	0.065	0.054	**0.042**	**0.042**

From the results in the last two tables, the RMSE values of the prediction models indicate that the data of the entire period provides the best overall results (the smallest mean error and minimum RMSE value). However, for a specific branch, such as C2 in Table 4, which was located close to a tourist attraction, splitting the data into regular and COVID-19 periods gave better results. This is perhaps because the number of local and international tourists decreased significantly after the outbreak of the CPVID-19 pandemic, causing the CO and CI values to decrease drastically. In addition to the results from the above tables, it was observed that in the COCI values during the regular and COVID-19 periods were not considerably different. This reveals that the differences between the CO and CI values, represented by the COCI values, remained the same regardless of the COVID-19.

The LSTM and GRU techniques were tested on various sets of data with additional features (v1 and v2) to find the most suitable approach for the cash stock prediction. Each dataset contains 30 days of CO and COCI data. The training losses were calculated by the RMSE values and were compared. The results revealed that LSTM provided lesser loss (measured in RMSE values) in most datasets. When consider the RMSE values of the LSTM and GRU methods on different branches as shown in Table 6, the number of times that LSTM performs better than GRU (denoted as number of winners) is higher. Thus, LSTM technique was selected for further experiments.

**Table 6:** LSTM and GRU comparison based on RMSE values of different branches in the 30-day period.

Prediction	RMSE							
	CO				COCI			
Algorithm	LSTM		GRU		LSTM		GRU	
Additional feature	v1	v2	v1	v2	v1	v2	v1	v2
Branch A1	**0.149**	**0.154**	0.162	0.169	0.086	**0.053**	**0.078**	0.076
Branch A2	**0.152**	**0.158**	0.166	0.165	**0.090**	**0.097**	0.110	0.105
Branch A3	**0.127**	**0.129**	0.146	0.140	**0.103**	**0.100**	0.135	0.138
Branch A4	**0.129**	**0.133**	0.149	0.151	**0.086**	**0.077**	0.106	0.104
Branch B1	**0.118**	**0.129**	0.140	0.136	**0.042**	**0.042**	0.064	0.066
Branch B2	**0.130**	**0.127**	0.139	0.140	**0.099**	**0.087**	0.100	0.104
Branch C1	**0.167**	**0.176**	0.173	0.182	**0.149**	0.155	0.156	**0.152**
Branch C2	**0.201**	0.212	0.233	**0.207**	**0.063**	**0.095**	0.101	0.099
Branch C3	**0.118**	**0.120**	0.128	0.125	**0.059**	**0.056**	0.072	0.070
Branch C4	**0.135**	0.143	0.146	**0.141**	**0.072**	0.127	0.123	**0.124**
Number of winners	**10+8 = 18**		0+2 = 2		**9+8 = 17**		1+2 = 3	

We have conducted additional experiments with Rectified Linear Unit (ReLU) and sigmoid functions as the activation functions to compare their performances. The results are shown in Tables 7 and 8. The tables list the RMSE results of CO and COCI predictions by using LSTM with different activation functions. The RMSE measures the deviation between the CO or COCI predictions against the actual values. Four comparisons were conducted: CO-v1, CO-v2, COCI-v1, and COCI-v2. The best activation functions in these comparisons are shown in a bold font. From the table, it was obvious that tanh produced the best overall results in terms of the number of times it yielded the least RSME among the three activation functions in these 10 branches. It should be noted that under CO-v2 comparison tanh and sigmoid gave the least RMSE for branch A2. In this case, both activation functions are counted as the best.

**Table 7:** RMSE results of CO predictions by using LSTM with different activation functions.

Algorithm	LSTM					
Prediction	CO					
Activation function	tanh		ReLU		sigmoid	
Additional feature	v1	v2	v1	v2	v1	v2
Branch A1	**0.149**	**0.154**	0.159	0.162	0.164	0.159
Branch A2	**0.152**	**0.158**	0.167	0.264	0.159	**0.158**
Branch A3	**0.127**	**0.129**	0.144	0.139	0.139	0.137
Branch A4	**0.129**	**0.133**	0.149	0.144	0.140	0.139
Branch B1	**0.118**	0.129	0.139	0.134	0.126	**0.113**
Branch B2	0.130	**0.127**	**0.128**	0.140	0.132	0.134
Branch C1	**0.167**	0.176	0.175	0.195	0.172	**0.165**
Branch C2	0.201	0.212	0.225	0.235	**0.155**	**0.151**
Branch C3	0.118	0.120	0.126	0.125	**0.106**	**0.109**
Branch C4	**0.135**	0.143	0.147	0.147	0.137	**0.136**
Number of winners	7+5 = **12**		1+0 = 1		2+6 = 8	

**Table 8:** RMSE results of COCI predictions by using LSTM with different activation functions.

Algorithm	LSTM					
Prediction	COCI					
Activation function	tanh		ReLU		sigmoid	
Additional feature	v1	v2	v1	v2	v1	v2
Branch A1	0.086	**0.053**	0.075	0.071	**0.071**	0.067
Branch A2	**0.090**	**0.097**	0.109	0.391	0.391	0.099
Branch A3	**0.103**	**0.100**	0.135	0.137	0.137	0.127
Branch A4	**0.086**	**0.077**	0.106	0.115	0.115	0.088
Branch B1	**0.042**	**0.042**	0.060	0.064	0.064	0.079
Branch B2	0.099	**0.087**	**0.093**	0.100	0.100	0.092
Branch C1	**0.149**	0.155	0.156	0.178	0.178	**0.147**
Branch C2	**0.063**	0.095	0.104	0.101	0.101	**0.062**
Branch C3	**0.059**	0.056	0.069	0.071	0.071	**0.055**
Branch C4	**0.072**	0.127	0.123	0.126	0.126	**0.114**
Number of winners	8+6 = **14**		1+0 = 1		1+4 = 5	

### Error estimation

The CO and COCI predictions were evaluated against the actual CO and COCI amounts at individual branches. The error measure was based on Eq (10). These errors were then tested against different distributions to determine those that best fit the error data. Three distributions that often appeared as good candidates were normal distribution, Cauchy distribution, and gamma distribution. Tables 9 and 10 summarize the distribution fitting results based on a chi-squared test for CO and COCI values, respectively. The two tables display the chi-squared value (χ^2^) and the corresponding p value of each distribution for each branch. For example, in Table 9 the chi-squared and p values of the normal distribution for Branch A1 are 1.15 × 10^−7^ and 1.00, orderly.

**Table 9:** Distribution fitting results of cash out (CO) prediction errors.

Branch	Normal Distribution		Cauchy Distribution		Gamma Distribution	
	χ^2^	p value	χ^2^	p value	χ^2^	p value
A1	1.15E-07	1.00E+00	3.05E-07	1.00E+00	1.13E-07	1.00E+00
A2	8.81E-07	1.00E+00	2.44E-06	1.00E+00	8.10E-07	1.00E+00
A3	6.37E-07	1.00E+00	1.80E-06	1.00E+00	6.50E-07	1.00E+00
A4	1.54E-06	1.00E+00	4.58E-06	1.00E+00	1.49E-06	1.00E+00
B1	1.48E-06	1.00E+00	1.89E-06	1.00E+00	6.56E-07	1.00E+00
B2	7.48E-07	1.00E+00	3.66E-06	1.00E+00	7.86E-07	1.00E+00
C1	2.90E-06	1.00E+00	7.46E-07	1.00E+00	NA	NA
C2	4.82E-06	1.00E+00	5.40E-06	1.00E+00	5.62E-06	1.00E+00
C3	7.83E-07	1.00E+00	1.53E-06	1.00E+00	6.72E-07	1.00E+00
C4	3.40E-07	1.00E+00	3.36E-07	1.00E+00	2.36E-07	1.00E+00

**Table 10:** Distribution fitting results of (COCI) prediction errors.

Branch	Normal Distribution		Cauchy Distribution		Gamma Distribution	
	χ^2^	p value	χ^2^	p value	χ^2^	p value
A1	7.04E-08	1.00E+00	1.28E-07	1.00E+00	4.71E-08	1.00E+00
A2	7.62E-07	1.00E+00	2.51E-06	1.00E+00	8.28E-07	1.00E+00
A3	1.15E-06	1.00E+00	2.69E-06	1.00E+00	9.04E-07	1.00E+00
A4	6.27E-07	1.00E+00	2.12E-06	1.00E+00	6.24E-07	1.00E+00
B1	3.75E-06	1.00E+00	8.73E-07	1.00E+00	NA	NA
B2	6.24E-07	1.00E+00	1.93E-06	1.00E+00	6.08E-07	1.00E+00
C1	8.55E-07	1.00E+00	1.18E-06	1.00E+00	4.73E-07	1.00E+00
C2	1.13E-06	1.00E+00	4.43E-06	1.00E+00	9.95E-07	1.00E+00
C3	7.42E-07	1.00E+00	1.90E-06	1.00E+00	7.32E-07	1.00E+00
C4	2.13E-07	1.00E+00	7.16E-07	1.00E+00	1.90E-07	1.00E+00

**Fig 8:**
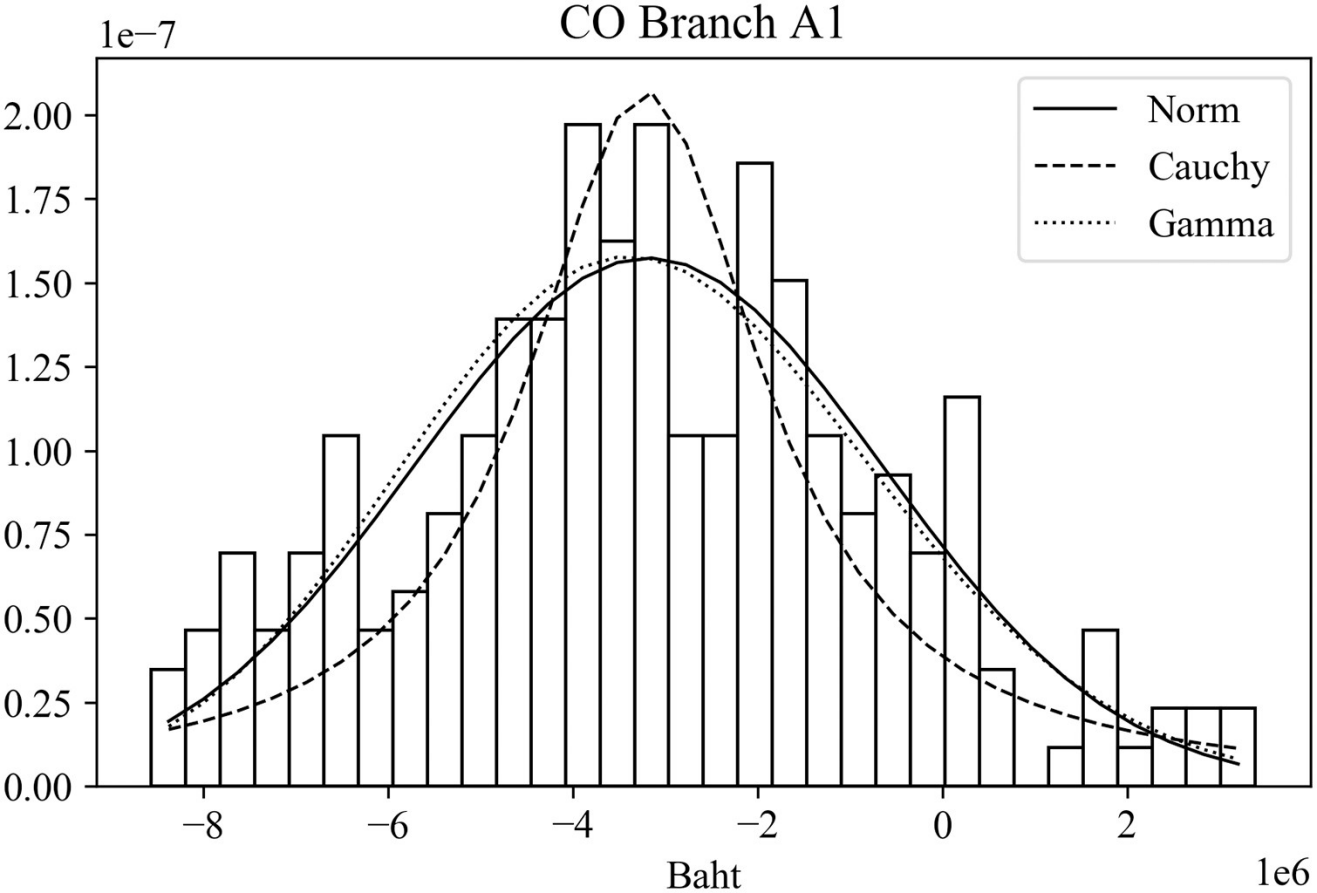
Examples of error distribution fitting results of CO.

**Fig 9:**
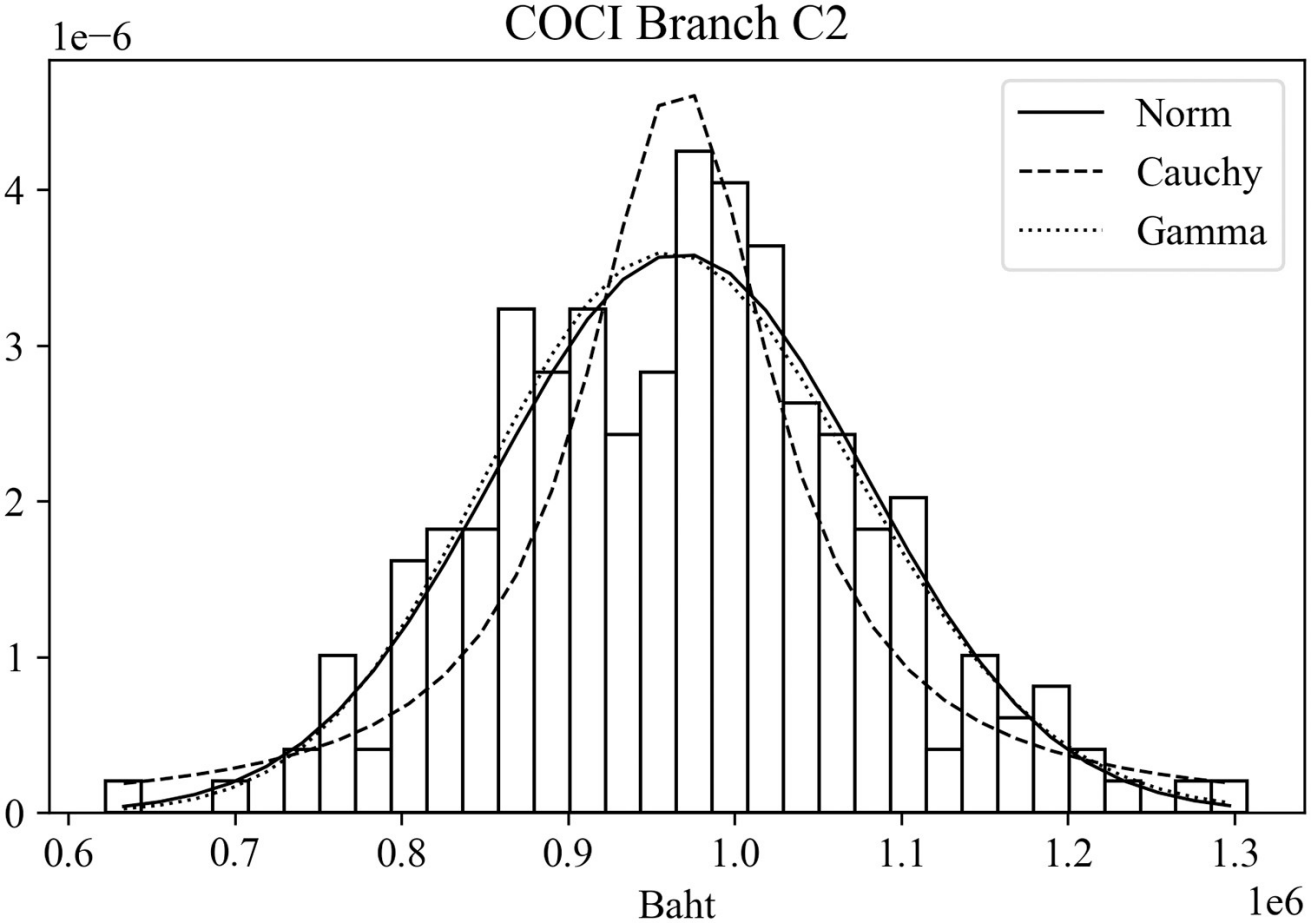
Examples of error distribution fitting results of COCI.

When comparing the chi-squared values of the three distributions of the same branch, it was found that normal and gamma distributions often gave better or at least comparable results to those of Cauchy distribution. Note that a small chi-squared value implies a good fit to the distribution. Figs 8 and 9 depict typical distribution fitting results of the error data. Fig 8 shows the probability density function of the CO errors for Branch A1 with the results of distribution fitting by the three distributions. Likewise, Fig 9 displays the results of Branch C2 based on the COCI error data. The horizonal axis of the figure represents the magnitude of the prediction errors. In Fig 8, the peak of all distributions situates on negative numbers. This means that for this branch the actual CO values are smaller than the predicted values. On the contrary, in Fig 9, the actual COCI values are greater than those that are predicted.

Reconsidering Tables 9 and 10 for Branch C1 in the CO case and Branch B1 in the COCI case, the gamma distribution did not fit well with the error data (the Python code did not give a numeric result) as indicated by NA (not applicable). These results inform us that the gamma distribution is not suitable to represent the prediction errors to these branches. As for the rest of the tables, the p values reveal that these distributions are statistically sound (at the significant level of 0.05) to represent the prediction errors. After consulting with the bank staff, it was decided to select the normal distribution as the sole representative of the prediction errors for ease of application as multiple distributions may lead to confusion when the method is implemented. Tables 11 and 12 summarize the means and standard deviations of these normal distributions for the CO and COCI cases, respectively. For both cases, additional experiments were performed to test if COVID-19 affect the errors. From the results, the pandemic does not seem to impact the mean and standard deviation of the normal distributions at different degrees. As for the bank, the main interest is how COVID-19 changes the cash safety stock or the $F_{E}^{-1}(1-\alpha )$ value. The results in Tables 11 and 12 indicate that with a few exceptions the cash safety stock required during the COVID-19 period is smaller or sometimes relatively similar to that of the same branch during the regular period. This may be because some customers migrate to online services to reduce physical contacts, or some increase the amount of cash per transaction to reduce the number of trips to the bank. For Branch A1 which is one of the flagship branches of the bank, the cash withdrawals seem to increase during the COVID-19 period as shown in Table 11. But the cash deposits of this branch also increase during the pandemic period. These deposits level out the cash withdrawal effect such that the cash stock requirements during both the regular and COVID-19 periods become similar as shown in Table 12.

**Table 11:** Parameters of the normal distribution of the CO prediction errors and the inverse of their cumulative distribution at *α* = 0.05.

Branch	Regular period			COVID-19 period		
	Normal distribution		Safety stock	Normal distribution		Safety stock
	Mean	SD		Mean	SD	
A1	-3.48E+06	2.97E+06	1.41E+06	-3.18E+06	2.53E+06	9.86E+05
A2	-3.90E+05	3.72E+05	2.22E+05	-3.00E+05	2.70E+05	1.44E+05
A3	-3.33E+05	5.62E+05	5.91E+05	-4.40E+05	4.57E+05	3.12E+05
A4	-4.01E+05	3.63E+05	1.96E+05	-1.18E+05	3.05E+05	3.84E+05
B1	-4.39E+05	5.88E+05	5.28E+05	-7.82E+05	4.47E+05	-4.59E+04
B2	-1.02E+05	2.79E+05	3.57E+05	-1.10E+05	2.57E+05	3.13E+05
C1	-7.01E+05	1.26E+06	1.38E+06	-6.18E+05	1.69E+06	2.16E+06
C2	-1.40E+04	2.60E+05	4.14E+05	-1.31E+06	8.96E+04	-1.16E+06
C3	1.50E+05	7.89E+05	1.45E+06	-6.88E+05	4.82E+05	1.05E+05
C4	-8.68E+05	1.30E+06	1.26E+06	-3.17E+06	1.50E+06	-7.11E+05

**Table 12:** Parameters of the normal distribution of the COCI prediction errors and the inverse of their cumulative distribution at *α* = 0.05.

Branch	Regular period			COVID-19 period		
	Normal distribution		Safety stock	Normal distribution		Safety stock
	Mean	SD		Mean	SD	
A1	5.79E+06	8.25E+06	1.94E+07	3.55E+06	5.76E+06	1.30E+07
A2	4.71E+04	4.11E+05	7.24E+05	2.12E+04	3.21E+05	5.50E+05
A3	-1.32E+05	8.71E+05	1.30E+06	1.72E+05	7.33E+05	1.38E+06
A4	5.32E+05	6.36E+05	1.58E+06	-5.61E+04	4.81E+05	7.35E+05
B1	2.08E+05	8.76E+05	1.65E+06	-8.35E+04	7.41E+05	1.14E+06
B2	-1.41E+05	6.84E+05	9.85E+05	-2.56E+05	6.48E+05	8.10E+05
C1	5.77E+05	1.00E+06	2.23E+06	2.04E+05	1.03E+06	1.90E+06
C2	1.00E+05	3.57E+05	6.86E+05	9.67E+05	1.11E+05	1.15E+06
C3	3.43E+05	8.60E+05	1.76E+06	2.49E+05	5.01E+05	1.07E+06
C4	3.20E+05	1.33E+06	2.51E+06	-2.03E+06	1.12E+06	-1.78E+05

The distributions of the errors allow us to integrate the prediction values into the cash stocking strategies. First, to limit the risk from prediction errors, the cash safety stock (SS) should at least equal to the value calculated from Eq (4). Using the risk level (*α*) of 0.05, the cash SS of all the branches are shown in Tables 11 and 12. When the SS values are positive, the results suggest that the bank should secure at least those amounts of cash as the safety net for the corresponding branches. But the negative SS values requires further interpretation. The negative SS values indicate that the prediction values are greater than the actual ones. If the bank is to rely on the prediction value to stock the cash, it simply does not need further cash as a safety stock, or it can set SS = 0 when the calculated SS is negative.

The cash SS suggested in Tables 11 and 12 covers only 95% of the risk. Theoretically, it is not possible to guard against all the risk because based on the normal distribution it requires an infinite amount of cash to be kept in stock to have an absolute zero risk. Nevertheless, for additional caution the bank decides to integrate these prediction errors with other measures to determine the final cash stock amount for individual branches as detailed in the next section.

### Expected cash stock prediction results

The deep learning model was trained and tested with the historical data coving both regular and COVID-19 periods. The learning model was applied to predict CO and COCI for 14 consecutive days using the previous 30 days of data in a rolling time window. The 14 consecutive days coincide with the planning horizon for the cash replenishment transportation schedule of the bank.

**Fig 10:**
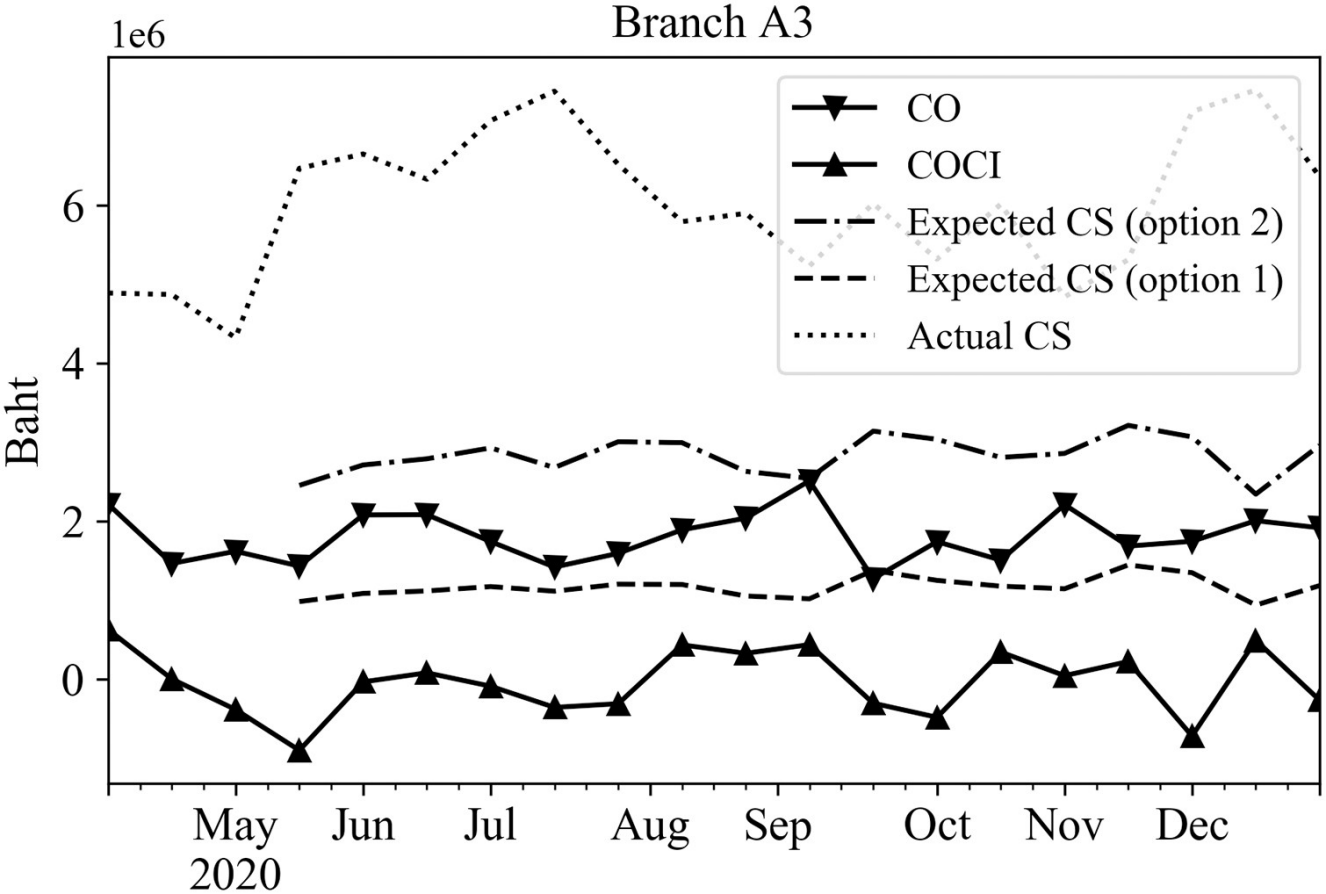
Expected and actual cash stocks, CO, and COCI of an example branch that opens every day.

**Fig 11:**
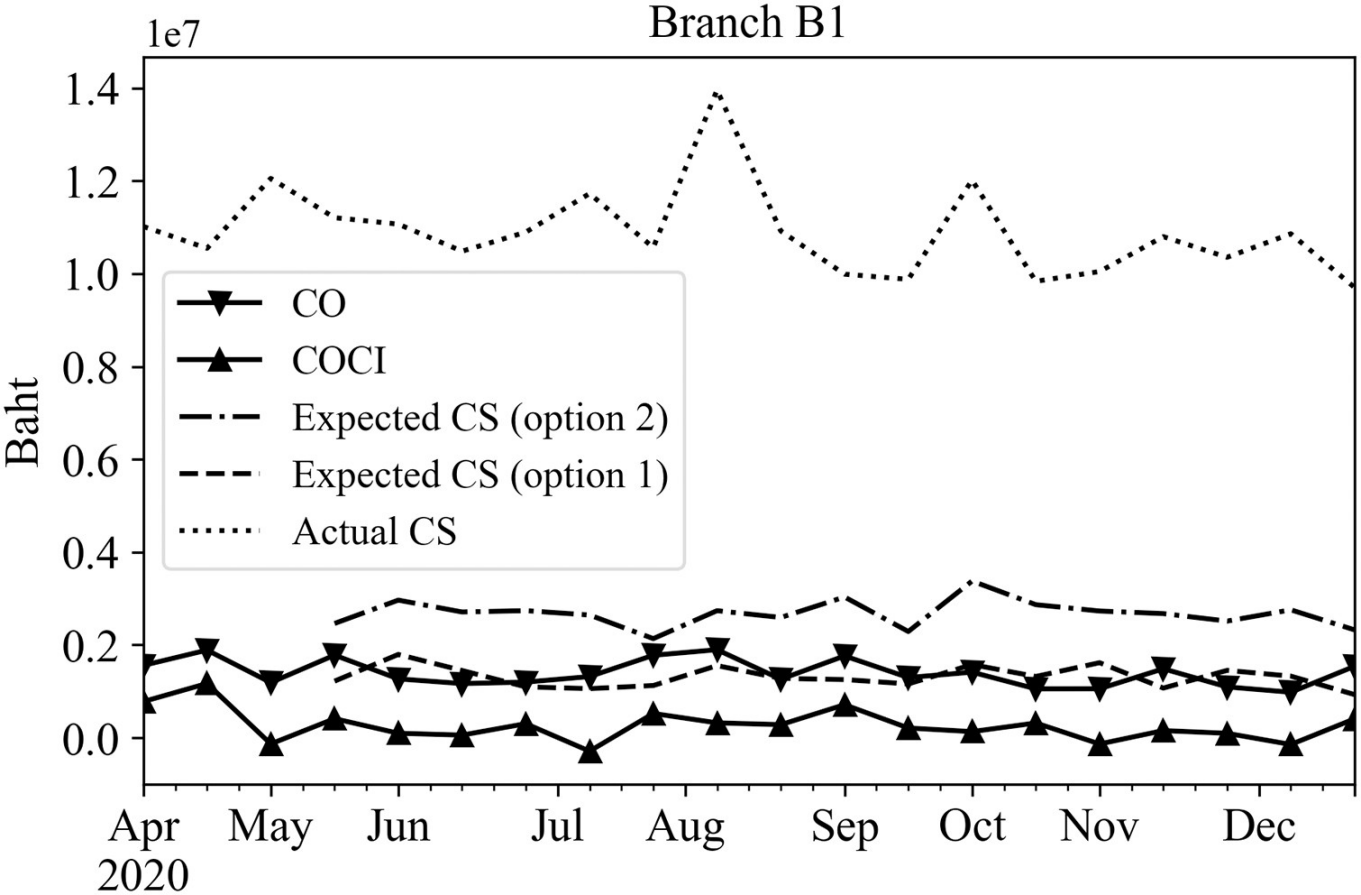
Expected and actual cash stocks, CO, and COCI of an example branch that opens Monday—Saturday.

**Fig 12:**
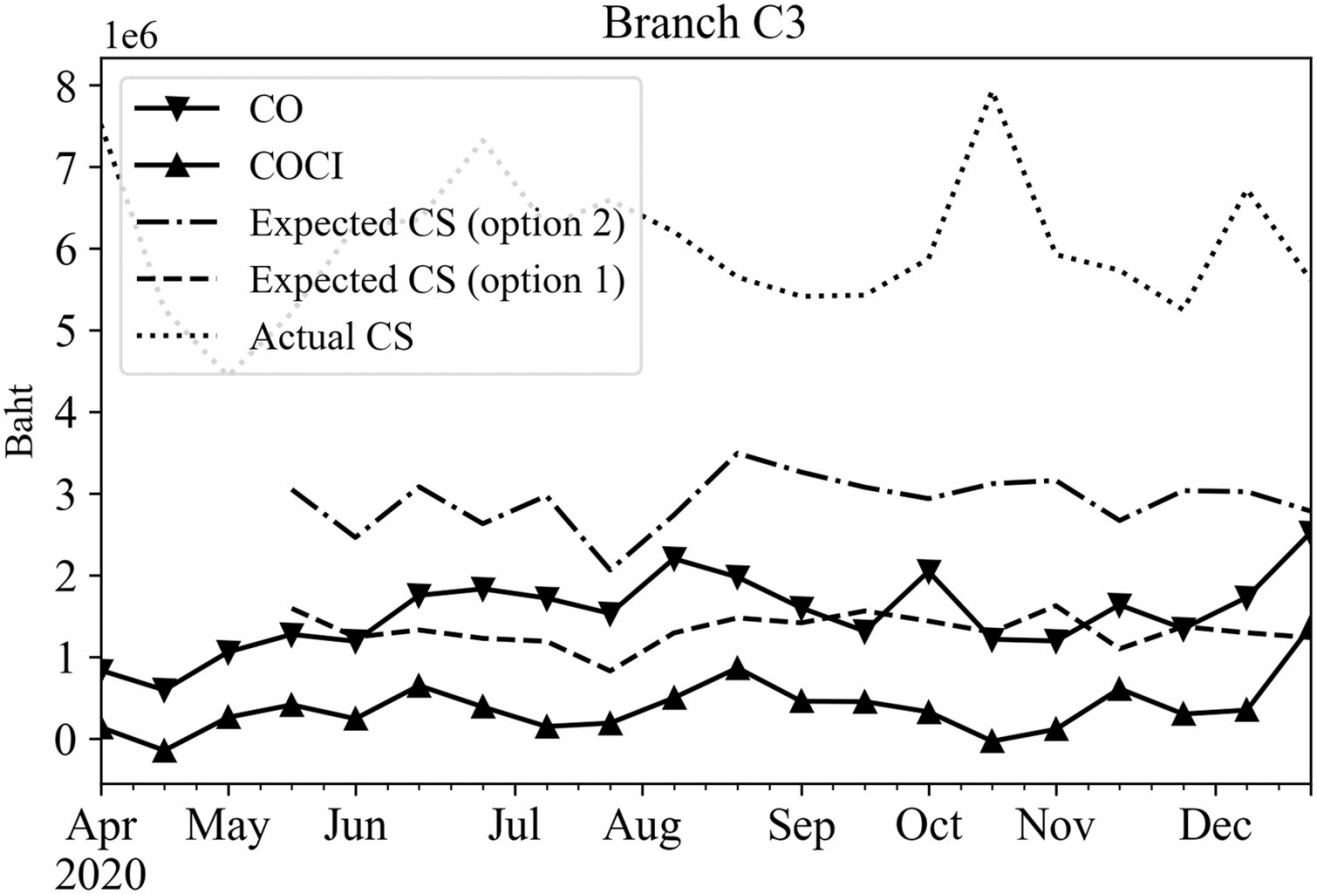
Expected and actual cash stocks, CO, and COCI of an example branch that opens Monday—Friday.

To represent the 14-day results as a single data point, the total predicted amounts of CO or COCI for the 14 days were summed together to a single value for each rolling time window. These predicted results were then used in Eqs (5) and (6) to determine the upper bound and lower bound values, respectively. These bounds were used in Eqs (7) and (8) to obtain the expected cash stock options 1 and 2 for the planning horizon of 14 days, orderly. Figs 10–12 show the expected cash stock prediction results of both options (dash and dot-dash lines) against the actual cash stock (dot line) as well as the actual CO (solid line with inverted triangles) the actual COCI (solid line with upward triangles) data. Since the actual CO and COCI values reflect cash withdrawal and deposit behavior of the customers, they should be treated as guidelines to set the cash stock level. These values were used to compare the expected cash stock via options 1 and 2. Figs 10–12 show examples of the actual CO or COCI, actual cash stock, and the expected cash stock using options 1 and 2 for three categories of branches according to their working days; Every day, Monday-Friday, and Monday-Saturday. The expected cash stock from option 2 generally provides sufficient cash amount to serve the maximum CO demands in all branch categories. The expected cash stock from option 1 provides a lower level of the cash stock than option 2. Even though, the cash stock level guided by option 1 may be riskier than that of option 2, it still covers the COCI values which represent the net cash withdrawals minus deposits.

The cash stock levels obtained through Eqs (7) and (8) are significantly lower than the level set by the branch manager (shown as ‘Actual Cash Stock’ line) in all categories of branches as shown in Figs 10–12. This shows that the cash stock levels set by options 1 and 2 could lower the cash stock levels of the branch, and face only limited statistically sound risk.

Tables 13 and 14 show the amounts of cash stock that could be reduced from the actual cash stocks during the regular and entire periods, respectively. The results show that the predicted cash stocks during the regular and entire periods provide almost similar results on average. Therefore, the data set for the entire period were used to train and test the model.

**Table 13:** Amount of cash stock of various options and 10% reduction target using the regular period.

Branch	Option1 (%)	Option1 (Baht)	Option2 (%)	Option2 (Baht)	10% reduction target of actual cash stock (Baht)
A1	89.95	63,217,200.00	77.05	54,153,360.00	7,027,986.00
A2	77.36	4,488,732.00	50.64	2,938,414.00	580,225.40
A3	81.83	5,337,894.00	55.54	3,623,235.00	652,306.80
A4	88.80	6,295,105.00	72.69	5,152,667.00	708,874.20
B1	86.76	9,367,680.00	69.53	7,507,419.00	1,079,724.00
B2	92.69	6,397,915.00	81.75	5,643,178.00	690,269.70
C1	74.32	10,732,670.00	39.46	5,698,053.00	1,444,085.00
C2	93.46	12,660,430.00	83.65	11,331,110.00	1,354,665.00
C3	74.75	5,250,090.00	48.77	3,425,494.00	702,322.20
C4	51.71	6,084,163.00	5.32	625,582.80	1,176,649.00
Average	81.16	12,983,187.90	58.44	10,009,851.28	1,541,710.73

**Table 14:** Amount of cash stock of various options and 10% reduction target using the entire period data.

Branch	Option1 (%)	Option1 (Baht)	Option2 (%)	Option2 (Baht)	10% reduction target of actual cash stock (Baht)
A1	93.36	60,774,260.00	84.19	54,807,980.00	6,509,862.00
A2	85.26	5,234,901.00	66.09	4,057,960.00	614,004.80
A3	78.84	4,850,380.00	51.49	3,167,755.00	615,237.90
A4	92.85	7,904,073.00	83.56	7,112,751.00	851,245.50
B1	88.56	9,687,934.00	76.07	8,321,989.00	1,093,947.00
B2	91.52	5,236,960.00	78.81	4,509,304.00	572,206.40
C1	68.55	8,477,829.00	34.20	4,229,804.00	1,236,647.00
C2	95.33	13,962,700.00	88.32	12,936,260.00	1,464,700.00
C3	79.32	4,883,211.00	52.37	3,224,147.00	615,602.90
C4	51.98	5,520,630.00	7.79	826,935.90	1,061,970.00
Average	82.56	12,653,287.80	62.29	10,319,488.59	1,463,542.35

During the regular period, the average reductions of the cash stock from options 1 and 2 were 12,983,187.90 Baht (81.16%) and 10,009,851.28 Baht (58.44%), respectively. During the entire period, the average reductions of the predicted cash stock from options 1 and 2 were 12,653,278.80 Baht (82.56%) and 10,319,488.59 Baht (62.29%), respectively. The increase for the entire period could be because some customers probably migrated to online services after the outbreak. Yet the branch manager seemed to set the cash stock level like when there was no pandemic. Both options give better results than 10% reduction which was initially set as the target to reduce the cash stock level (as shown in the last column of Tables 13 and 14).

### Managerial implication

The bank was more comfortable to carefully and gradually adjust its cash stock strategies. The 10% reduction in the cash stock level is to be adopted for the next few years to demonstrate that this policy is achievable. If the policy is proven to be practical and reliable, then the expected cash stock would be deployed with parameter setting recommended by the management. The approach presented here has exceeded the goal of reducing the cash management at the branch, and more importantly is a helpful tool to support the branch manager in operation decisions.

To apply the approach proposed in this research to other banks, certain cautions are worth mentioning. First, the 14-day planning horizon should be reconsidered to fit the need of the planner. Of course, a shorter planning horizon always gives more accurate results than a long one. However, the 14-day predictions in this research follow the planning interval of cash transportation of the bank. The tested models reported in this research were merely those that gave relatively good results. The rest of the models was not reported, and was executed with other variations of the input features. It is a common practice that many models are to be tested on different input features, and different periods of data to select only a few models with the best performances to implement. The available data are dynamic as new data arrive, so should the prediction models. It is possible that the model that best fits the available data today may perform worse later with the updated data. Therefore, the model, especially its input parameters, should be occasionally retrained. A signal to re-evaluate the prediction model is when its RMSE value substantially increases. Consequently, the distribution of the prediction errors should be re-examined when the prediction model is altered. The last issue is computational resources. The tests in this research were sequentially performed on personal computers and it took several hours to complete. This could be opted to a cloud service if the approach is to be executed for the rest of the branches, even though it may also impact how often the model, parameters, and error distributions are to be updated.

## Conclusion

The objective of this research is to set the cash stock level at individual bank branches from the available historical data instead of relying solely on the experience of the branch managers. Although the data reflect the behavior of the customers, other managerial concerns must also be addressed. Cash withdrawals and deposits, of course, affect the cash stock level of the branch, and precaution measures must be considered to determine a suitable cash stock level. To do so, the research methodology here was divided into three main phases: cash prediction modelling which included only cash out as well as both cash out minuses cash in, error estimation to find the cash safety stock as a safeguard against prediction errors and expected cash stock calculation by which managerial precaution measures are integrated.

To determine the level of the cash stock, we first applied encoder and decoder techniques to transform the input structure to the output one as shown in Fig 6. The mathematics underpinning LSTM and GRU are based on the papers by [21, 22]. Second, several distribution models were statistically tested to estimate the prediction errors from LSTM to prevent the risk from directly adopting the predictions made by LSTM. Third, strategies to determine the level of cash stock based on lower and upper bounds were developed. These strategies were suggested by the bank personnel to ensure their practicality as illustrated in Eqs (7) and (8). The contribution of this study is in extension and application of the deep learning approach to cash stock prediction, as well as addressing practicality in a real-world setting.

In the cash prediction modelling phase, the LSTM technique was applied to the cash withdrawals for which the cash demands, referred as cash out (CO), was the primary consideration. The technique was also carried out for the net cash demands by subtracting the cash deposits from the cash withdrawals, denoted by cash out minuses cash in (COCI). If the total amount withdrawn exceeded the total amount deposited resulting in a positive net cash out minuses cash in, the on-hand cash stock depleted, and the branch manager would request cash replenishment. On the contrary, if the net cash out minuses cash in was negative, it meant that the total cash deposited surpassed the total cash withdrawn. This raised the cash stock level, and the branch manager could either keep the additional cash or order it to be transported to the central cash center to maintain the cash stock at the predetermined level. Normally, each branch manager would assess these cash withdrawals and deposits and reacted based on his/her experience.

The LSTM technique was tested by varying its input parameters which were the input features and time periods to generate six different test models. It was found that the input feature version 1 activated by the tanh function and using the entire period data provided the best overall results in terms of the RMSE values for both CO as well as COCI in the models. However, for better results in some of the branches separating the data into regular and COVID-19 would be a better option. Both CO and COCI prediction models via the LSTM technique were later combined into the same computer code with common input features to save computational time.

In the error estimation phase, three distribution models often appeared to fit well with the prediction errors based on the chi-squared test results. The normal distribution yielded a comparable or better performances than the Cauchy distribution did to the same set of the prediction error data, while the gamma distribution did not find numerical results in some of the branches. Hence, the normal distribution was chosen to describe the errors from the prediction. The prediction errors imply the risk associated with the prediction values if they were to be used to estimate the withdrawals and deposits. The bank accepted the risk level due to the prediction errors (α) at 0.05. The amount of cash associated with this risk level was calculated and treated as the cash safety stock for each of the branches. If this amount was a negative number, it was set to zero.

The prediction and safety stock together with managerial concerns were considered in setting the cash stock level in the expected cash stock phase. Two options were proposed. Option 1 was computed based on CO and COCI. However, the bank could choose different values of *θ* and *r*_1_ that best suit its cash management strategies. Option 2 was more conservative as it was relying primarily on the upper bound of the cash stock level and the parameter *r*_2_. The expected cash stocks computed by option 2 were generally higher than those found by option 1. The bank could adopt option 2 if it is willing to sacrifice the profit that could be made by the cash exceeding that in option 1.

To validate the performance of our proposed approach, the data of ten branches were tested and compared to the actual cash stock levels deployed by the bank managers. The test was performed on test data of different periods: regular, COVID-19, and entire periods. To obtain the best overall prediction, the data from the entire period should be utilized for most branches. However, for better results of some branches the trained model with separated data sets—regular and COVID-19 periods—are preferred.

The average saving of the 10% target was approximately 1.4 to 1.5 million Baht per day for each branch tested. To achieve this target, our proposed model with option 1 attributes is selected as it provides the total saving over 400 million Baht a day for all the 628 branches of the bank. This alone could greatly reduce the insurance premium of the bank.

## Supporting information

S1 Table Attribute description. (DOCX)
