# Supplementary material for: Cash stock strategies during regular and COVID-19 periods for bank branches by deep learning
Source: PLoS One. 2022 Jun 7;17(6):e0268753. doi: 10.1371/journal.pone.0268753 (PMC9173617; doi:10.1371/journal.pone.0268753)
Supplement: S1 Table — (DOCX) [file pone.0268753.s001.docx]

# Supporting Information

**S1 Table Attribute description**

| Attribute names | Description |
| --- | --- |
| EFFECTIVE_DATE | Date the bank received data before 23.59 |
| BRANCH_CODE | Code of the branch |
| BRANCH_NAME | Name of the branch |
| CASHIN_AMT | Amount of cash deposited by customers that is less than 1 million baht per transaction |
| CASH_OUT_AMT | Amount of cash withdrawn by customers that is less than 1 million baht per transaction |
| CASH_IN_AMT_GT1M | Amount of cash deposited by customers that is greater than or equal to 1 million baht per transaction |
| CASH_OUT_AMT_GT1M | Amount of cash withdrawn by customers that is greater than or equal to 1 million baht per transaction |
| SHIPIN1K | Amount of cash requested by branch to be shipped in from the cash centre in the form of 1000-baht denomination |
| SHIPIN500 | Amount of cash requested by branch to be shipped in from the cash centre in the form of 500-baht denomination |
| SHIPIN100 | Amount of cash requested by branch to be shipped in from the cash centre in the form of 100-baht denomination |
| SHIPIN_OTHER | The amount of cash requested by branch to be shipped in from the cash centre in the form of coins and other denominations |
| SHIPOUT1K | Amount of cash shipped out to the cash centre in the form of 1000-baht denomination |
| SHIPOUT500 | Amount of cash shipped out to the cash centre in the form of 500-baht denomination |
| SHIPOUT100 | Amount of cash shipped out to the cash centre in the form of 100-baht denomination |
| SHIPOUT_OTHER | The amount of cash shipped out to the cash centre in the form of coins and other denominations |
| CASHSTOCK1K | Amount of cash remained at the end of the day in the form of 1000-baht denomination |
| CASHSTOCK500 | Amount of cash remained at the end of the day in the form of 500-baht denomination |
| CASHSTOCK100 | Amount of cash remained at the end of the day in the form of 100-baht denomination |
| CASHSTOCK_OTHER | The amount of cash remained at the end of the day in the form of coins and other denominations |
